# Prevalence and risk factors for bone loss in rheumatoid arthritis patients from South China: modeled by three methods

**DOI:** 10.1186/s12891-021-04403-5

**Published:** 2021-06-12

**Authors:** Zhuoran Hu, Lei Zhang, Zhiming Lin, Changlin Zhao, Shuiming Xu, He Lin, Jiejing Zhang, Wenjie Li, Yongliang Chu

**Affiliations:** 1 Division of Rheumatology, the Third Affiliated Hospital of Sun Yat-sen University, No.600, Tianhe Road, Tianhe District, Guangzhou City, 51000 China; 2 Division of Rheumatology, Zhuhai Hospital of Guangdong Provincial Hospital of Chinese Medicine, No.53, Ji’Da Jingle Road, Xiangzhou District, Zhuhai City, 519015 China; 3 Division of Cardiology, the Third Affiliated Hospital of Sun Yat-sen University, No.600, Tianhe Road, Tianhe District, Guangzhou City, 51000 China; 4 Division of Rheumatology, Ganzhou Municipal Hospital, No.49, Dagong Road, Ganzhou City, 341000 China; 5 Division of Rheumatology, Fujian Provincial Hospital, No. 134, Dongjie Road, Fuzhou City, 350000 China

**Keywords:** Arthritis, rheumatoid, Osteoporosis, Prevalence, Risk factor

## Abstract

**Background:**

To explore the prevalence of bone loss among patients with rheumatoid arthritis (RA) and healthy controls (HC) and further explored the risk factors for osteopenia and osteoporosis of RA patients.

**Methods:**

A cross-sectional survey was undertaken in four hospitals in different districts in South China to reveal the prevalence of bone loss in patients. Case records, laboratory tests, and bone mineral density (BMD) results of patients were collected. Traditional multivariable logistic regression analysis and two machine learning methods, including least absolute shrinkage selection operator (LASSO) and random forest (RF) were for exploring the risk factors for osteopenia or osteoporosis in RA patients.

**Results:**

Four hundred five patients with RA and 198 HC were included. RA patients had lower BMD in almost BMD measurement sites than healthy controls; the decline of lumbar spine BMD was earlier than HC. RA patients were more likely to comorbid with osteopenia and osteoporosis (*p* for trend < 0.001) in the lumbar spine than HC. Higher serum 25-hydroxyvitamin D3 level and using tumor necrosis factor inhibitor in the last year were protective factors; aging, lower body mass index, and increased serum uric acid might be risk factors for bone loss.

**Conclusions:**

RA patients were more prone and earlier to have bone loss than HC. More attention should be paid to measuring BMD in RA patients aging with lower BMI or hyperuricemia. Besides, serum vitamin D and all three measurement sites are recommended to check routinely. TNFi usage in the last year might benefit bone mass.

**Supplementary Information:**

The online version contains supplementary material available at 10.1186/s12891-021-04403-5.

## Background

Rheumatoid arthritis (RA) is a chronic inflammatory autoimmune disease characterized by persistent synovitis and the progressive destruction of bones and cartilage in multiple joints [1]. Osteoporosis (OP) is a well-known extra-articular complication in patients with RA [2], except for pulmonary involvement, cutaneous manifestations, and cardiovascular disease. The disorder of tumor necrosis factor-α (TNF-α), a key proinflammatory cytokine in RA, can stimulate osteoclastogenesis via receptor activator of nuclear factor kappa B ligand (RANKL) activation. As a result, RA patients with OP increased the risk of osteoporotic fracture (OPF), an outcome that impairs quality of life and leads to mortality [3, 4]. The 1-year cumulative mortality rate due to hip OPF in RA patients was reported to be approximately 20% and significantly higher than that in general population [5]. Accordingly, appropriate management of OP and osteopenia for preventing fragility fracture in patients with RA are crucial to optimize clinical outcome [6].

The frequency of OP in patients with RA has been reported from 11 to 38.9% in the lumbar spine and from 6.3 to 36.2% in the total hip [3, 4, 7, 8], and the risk of developing into OP in RA patients are nearly twice compared with the general population [3]. Traditional risk factors, like age and low body mass index (BMI), and disease-specific risk factors, including glucocorticoids (GCs) treatment, immobilization, reduced physical activity due to tender joints, and muscle weakness, were frequently reported [9–12]. TNF inhibitor (TNFi), one of the representative biological disease-modifying anti-rheumatic drugs, has been reported either improvement or stable BMD among TNFi users in several prospective studies [13, 14].

However, insufficient information is available for the frequency of osteopenia and the distribution of bone mineral density (BMD) in three sites of BMD measurement in RA patients in China [15–17]. Therefore, we undertook a cross-sectional survey in four hospitals from different South China districts to explore the prevalence of bone loss and investigate BMD’s differences among patients with RA and healthy controls (HC). Meanwhile, we explored the risk factors for osteopenia and OP of RA patients, modeled with conventional logistic regression and another two machine-learning modeling methods to ensure robustness.

## Methods

### Patients

**Fig. 1:**
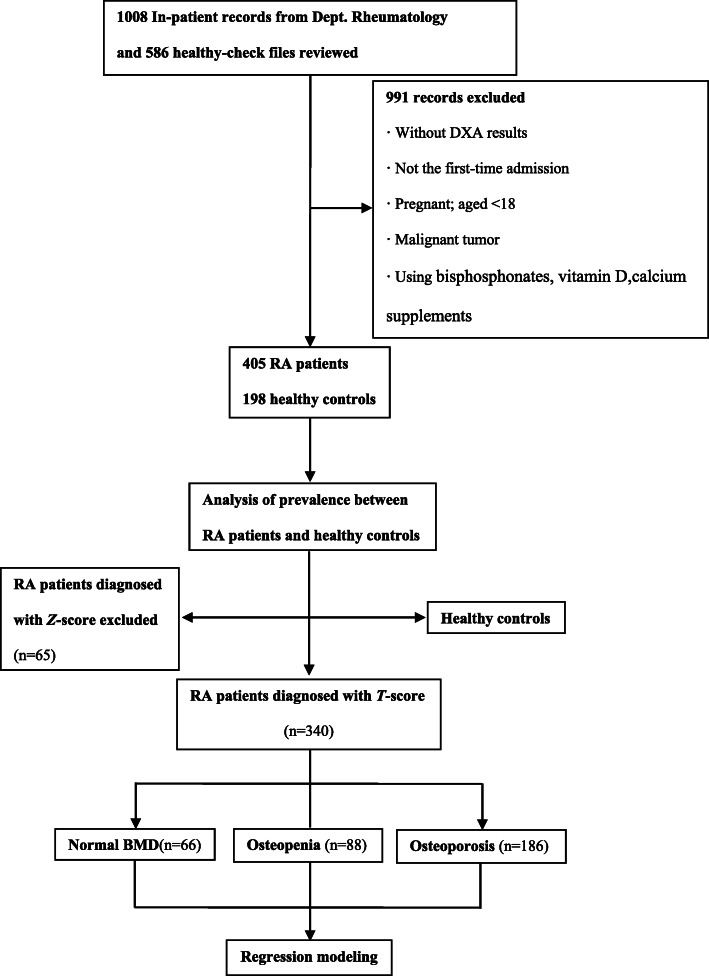
Study flow diagram. Diagnosed with *T*-score: for those are post-menopausal women and men aged ≥50. Diagnosed with *Z*-score: for those are pre-menopausal women and men aged < 50

We included the RA in-patients in four hospitals from October 2018 to August 2019 in the Third Affiliated Hospital of Sun Yat-sen University, Zhuhai Hospital of Guangdong Chinese Medicine, Ganzhou Municipal Hospital, and Fujian Provincial Hospital. The population of interest was 18 or older and diagnosed with RA (satisfied the 2010 ACR / EULAR classification criteria [18]). Age, gender-matched HC were contemporarily and randomly reviewed and selected from healthy-check files with DXA results in four centers in the same period. For RA patients and HC with more than one admission or record in the study period, only data from the first admission or record were analyzed. Exclusion criteria included when participants were unable to answer questions, pregnant, with parathyroid disorders, with a malignant tumor, refused to write informed consent or refused to have a dual-energy X-ray absorptiometry (DXA). The principal center was the Third Affiliated Hospital, Sun Yat-sen University. The detailed study flow diagram is shown in Fig. 1. Laboratory tests and DXA were done as parts of clinical routines.

### Main outcome variable

BMD, *T*-score, and the *Z*-score of the lumbar spine 2–4, femoral neck (right), and total hip (both sides) were collected from DXA reports (Hologic Discovery A densitometer, Badford, MA, USA) after blood samples had been taken. In our study, the diagnosis based on the results of BMD is the outcome variable. According to the World Health Organization [19], the definition of the *T*-score and the *Z*-score generates the results of BMD. A *T*-score ≥ − 1.0, between − 1.0 and − 2.5, and ≤ − 2.5 represent the expected condition, osteopenia, and osteoporosis, respectively, as a diagnosis standard for men aged and over 50 and postmenopausal women. Meanwhile, the *Z*-score is used for premenopausal women and males aged under 50. A *Z*-score of − 2.0 or lower indicates a lower BMD compared to the peers (‘score below the expected range for age’). Therefore, both HC and patients were divided into two subgroups (normal BMD or impaired BMD) regardless of age and menopausal status; then stratified these two subgroups into five ones by BMD results taking age and menopausal status into account, namely “score below the expected range of age” or normal BMD in premenopausal women and men aged < 50; “osteopenia”, “osteoporosis” and normal BMD in postmenopausal women and men aged > = 50.

### Covariates

Thirty-three independent variables (Table 1) were also collected, values belonged to the BMD measurement time. Smoking and drinking habits, medical and medication history, and laboratory examinations were taken from each participant’s history. Dyslipidemia included hypercholesteremia and hypertriglyceridemia. ‘Chronic usage’ of non-steroidal anti-inflammatory drugs (NSAIDs) or GC was defined as consecutively taking these medications at least the last 3 months. ‘Rheumatoid factor positive’ was defined when the concentration reached or over 30 IU/ml; anti-cyclic citrullinated peptide antibodies (anti-CCP), antikeratin antibodies (AKA), and anti-RA33 antibodies (RA33) ‘positive’ was defined when their concentrations were at or over 20 IU/ml. Insufficiency and deficiency (hypovitaminosis) of vitamin D were defined when serum 25-hydroxyvitamin D level is under 75 nmol/L (30 ng/ml) and 50 nmol/L (20 ng/ml) [20], respectively. Ethical approval was obtained from the Ethics Committee of the Third Affiliated Hospital of Sun Yat-sen University (Guangzhou, China). The registration no. of ethics approval of the study was [2018]02–283-01. Written informed consent was obtained from all individuals participating in this study.

**Table 1:** Candidate variables

Domains	Variables^a^
Demographics and lifestyles^b^	(1) age, (2) BMI, (3) gender, (4) smoker, always or never/seldom, (5) drinking, always or never/seldom
Medical history^b^	(6) hypertension, (7) diabetes mellitus, (8) coronary heart disease, (9) hyperuricemia, (10) dyslipidemia, (11) femoral neck necrosis
Medication history of RA patients^b^	(12) chronic NSAIDs usage, (13) chronic GC usage, (14) types of cDMARDs recently taking, (15) TNFi usage in the last one year, (16) overall anti-osteoporotic medication history^b^, (17) disease duration
Laboratory	(18) serum calcium level, (19) serum phosphate level, (20) sCr, (21) sUA, (22) CRP level^b^, (23) ESR^b^, (24) rheumatoid factor concentration^b^, (25) anti-CCP concentration^b^ (26) serum 25(OH)D3 level^b^, (27) C3 level, (28) C4 level, (29) CH50 level, (30) ANA titer, (31) rheumatoid factor positive, (32) anti-CCP positive, (33) AKA positive, (34) RA33 positive

*BMI* Body mass index, *NSAIDs* Non-steroidal anti-inflammatory drugs, *GC* Glucocorticoid, *cDMARDs* Conventional disease-modifying anti-rheumatic drugs, *TNFi* Tumor necrosis factor-α inhibitor, *sCr* Serum creatine level, *sUA* Serum uric acid level, *CRP* C-reactive protein, *ESR* Erythrocyte sedimentation rate, *anti-CCP* Anti-cyclic citrullinated peptide antibodies, *AKA* Antikeratin antibodies, *RA33* Anti-RA33 antibodies, *C3* Complement component 3, *C4* Complement component 4, *CH50* Serum total complement activity, *ANA* Antinuclear antibodies

^a^All variables were included in statistics-driven (LASSO) and random forest model. Except from variable 1,2,16–29, all variables were categorial or dichotomous

^b^Factors selected for the clinical knowledge-preselected model

### Sample size

The sample sizes were estimated by PASS 15 software (https://www.ncss.com, module ‘test for two proportions’), with the statistical power (1-β) set 0.90, type I error (α) set 0.05. Since HC data (total 200, 2 was omitted because their menopausal status was unknown) was collected first, we used the ‘Enter N1 solve for N2’ function to calculate the sample size of patients. Assuming that the prevalence of complicating with OP was 40% [8] among RA patients and was 23.2% among healthy Southern Chinese [21], the software eventually calculated that a sample size of RA patients was at least 149 would suffice. To ensure adequate events of each subgroup, we finally recruited 405 patients for the present study.

### Statistical analysis

Data were manually entered into EpiData (http://www.epidata.dk/) and then imported into Microsoft Office Excel (version 2016). Two physicians rechecked and transferred this data to the R software (version 3.6.1) for analysis. Continuous variables are marked as the mean ± standard deviation (SD), while discrete variables are presented as frequency and percentage. Dependent variables / primary outcomes were the T-score, Z-score, and corresponding diagnoses of BMD of the lumbar spine, femoral neck, and total hip, divided and stratified as mentioned above. A two-tailed t-test was used for comparing normally distributed continuous variables, and the *Kruskal-Wallis H* test was for non-normally distributed ones. *Pearson’s χ* ^*2*^ or *Fisher’s exact* test was performed for categorical variables and the Cochran-Armitage trend test for appropriate ordinal variables. R (version 3.6.1) was used for statistical analyses, and statistical significance was assumed at the *p* < 0.05 level.

### Model development

Owing to the inadequate amount of young RA patients, predictive models were only created for RA patients whose BMD was diagnosed with T-score. We took three different approaches of regression model development to ensure the robustness and validity of the regression models: clinical knowledge-driven, conventional logistic regression models (model A), least absolute shrinkage and selection operator (LASSO, model B), and random forest (RF, model C). We separated the data of all subgroups randomly into training sets (70%) and verification sets (30%), with the same positive-event proportion; the training set was for modeling, and the other was for validation, which could be evaluated by C-statistics, calibration slope, and the accuracy.

Model A: We preselected and then entered candidate variables based on existing literature or well-established risk factors into logistic regression models. The final set of variables included only those with a *p*-value< 0.05 from the regression analysis. ‘glm’ function in the basic package of R would be used.

Model B: LASSO is an ideal method to improve multicollinearity [22]. The LASSO procedure (especially for determining the optimal shrinkage estimator) underwent 5-fold cross-validation to avoid over-fitting. We entered all 34 candidate variables into the LASSO models. Package ‘glmnet’, function ‘cv.glmnet’ [23] would be used.

Model C: Random forest model assembles hundreds of more classification trees with a selection of correlates randomly [24]. We applied all 34 variables into the random forest models. The out-of-bag (OOB) estimates error rates; the Gini index was used to refer to the relative importance of the correlates after features were randomly permuted [25]. We identified the covariates by an increase in the Gini index by > 5. Package ‘randomForest’ would perform the modeling.

## Results

### Clinical features

Data of 405 patients with RA and 198 HC were included in the first step analysis. Missing data occurred only in part of DXA’s measurement sites; 12 of 198 healthy subjects (6.1%) included in the first step analysis, and 8 of 405 patients (2.0%) did not have DXA in the femoral neck (right) or total hip. The characteristics of the participants are presented in Table 2 and Supplementary Figure 1. All our participants were aged or over 40. The difference in gender composition (*p* = 0.256) and age (60.4 ± 10.4 vs. 59.4 ± 10.3, *p* = 0.275) between HC and patients was not significant. Although the BMI of HC was higher than those with RA (22.8 ± 3.62 vs. 22.1 ± 3.39, *p* = 0.031), the composition of BMI groups [26, 27] showed no significant difference. HC had more postmenopausal women and even those with early menopause than patients with RA (88.4% vs. 70.7%, *p* < 0.001). Serum calcifediol [25(OH)D3] level was lower in HC (65.6 ± 22.1 vs. 76.7 ± 32.9, *p* = 0.006); insufficiency of vitamin D3 and hypovitaminosis D were also more prevalent in RA patients (64.9% vs. 54.5%, *p* for trend test =0.046). The median disease duration of RA patients was 5.5[1.5;13.0].

**Table 2:** Characteristics of the participants

Characteristics	HC, ***n***==198	RA, ***n*** = 405	***p***
**Demographic**			
Age, years, mean (SD)	60.4 (10c.4)	59.4 (10.3)	0.275
Disease duration, years, median [IQR]	NA	5.5[1.5;13.0]	NA
BMI, Kg/m^2^, mean (SD)	22.8 (3.6)	22.1 (3.4)	0.031
Female, n (%)	146 (73.7)	317 (78.3)	0.256
Menopause status of female, n (%)			< 0.001*
Post-menopause, age > 45	104 (71.2)	186 (58.7)	
Early menopause, age ≤ 45	25 (17.1)	38 (12.0)	
**Lifestyle**			
Smoking, ever or current, yes, n (%)	17 (8.6)	36 (8.9)	0.937
Drinking, ever or current, yes, n (%)	18 (9.1)	18 (4.4)	0.035
**Medical history**			
Diabetes mellitus	35 (17.7)	47 (11.6)	0.055
Hypertension	75 (37.9)	77 (19.0)	< 0.001
Coronary heart disease	20 (10.1)	20 (4.9)	0.027
Hyperuricemia	15 (7.6)	43 (10.6)	0.297
Dyslipidemia	58 (29.3)	56 (13.8)	< 0.001
Femoral neck necrosis	1 (0.5)	8 (2.0)	0.268
Osteoporotic fracture	0	20 (4.9)	NA
Anti-osteoporotic medication	6 (3.0)	29 (7.2)	0.641
**Laboratory**			
Serum calcium level, mmol /L, mean (SD)	2.4 (0.2)	2.3 (0.1)	< 0.001
Serum phosphate level, mmol /L, mean (SD)	1.9 (8.1)	1.2 (0.4)	0.272
Serum creatinine level, μmol /L, mean (SD)	67.2 (32.9)	62.6 (20.5)	0.086
Serum Uric acid level, μmol /L, mean (SD)	327.9 (101.2)	314.1 (103.9)	0.146
Serum 25(OH)D3 level, nmol /L, mean (SD)	65.6 (22.1)	76.7 (32.9)	0.006
Vitamin D insufficiency, yes, n (%)	65 (32.8)	145 (35.8)	0.046*
Hypovitaminosis D, yes, n (%)	43 (21.7)	118 (29.1)	0.046*

*: *p* for trend with Cochran-Armitage test; *HC* Healthy controls, *RA* Rheumatoid arthritis, *hypovitaminosis D* Serum 25(OH)D3 < 50 nmol/L, *VitD3 deficiency* Serum 25(OH)D3 < 75 nmol/L

### Difference and changing trend of BMD

We divided age into five groups by 5 years, according to van Staa TP, et al. grouping method [28], for analyzing the difference and changing trend of BMD of each site between HC and RA patients. The detailed analysis showed that except for those were aged 40–45, RA patients in all age groups had lower BMD in lumbar spine 2–4 (supplementary Table 1). BMD of the femoral neck was consistently lower in patients with RA at all age stages. However, except for patients who were 40–45 and 56–60 years old, BMD of the total hip was significantly lower than HC.

The visually intuitionistic changing trends of BMD of 3 measurement sites were both fluctuating but overall declining with age (Supplementary Figure 3a). The numerically highest decline in the BMD of the lumbar spine was found in RA patients aged over 50. BMI was positively correlated to BMD in all measurement sites for both patients and HC (see supplementary Figure 3b).

### Prevalence of bone loss

Since participants in our study were mid-aged or older, only 93 people were diagnosed with Z-score. We did not find a significant difference between HC and RA patients in any BMD measurement site.

In male participants aged at and over 50 and postmenopausal women, we noticed that RA patients were more likely to comorbid with osteopenia (24.1% vs. 32.3%) and OP (48.8% vs. 57.3%, p for trend < 0.001) only in the lumbar spine. However, the prevalence of osteopenia and OP in any site, or femoral neck, or total hip showed no difference between cases and controls (Supplementary Table 2 and Supplementary Figure 2).

### Risk factors for bone loss in RA patients

The three modeling approaches for three sites resulted in 18 different sets of variables associated with osteopenia and OP (9 sets of each). Two variables were consistently selected across all models, ‘age’ and ‘BMI’ (except ‘BMI’ in model B of the lumbar spine in osteopenia). Details of models were shown in (Supplementary Table 3). Finally, for osteopenia, model A, Model B, and Model A were the best for the lumbar spine, femoral neck, and total hip, respectively. For OP, model A, model B, and model B were optimal for the lumbar spine, femoral neck, and total hip, respectively. Odd ratios of the selected models were shown in Table 3. Aging was a general risk factor for each site and osteopenia (OR: 1.11 ~ 1.17) and osteoporosis (OR: 1.15 ~ 1.25). On the contrary, increasing BMI was a common protective factor for BMD (for osteopenia, OR: 0.84–0.88; for OP, OR: 0.62–0.68). Higher serum 25(OH)D3 level was a protective factor for lumbar spine [for osteopenia, OR: 0.99(0.98–1.00); for OP, 0.97(0.96–0.98)] and osteoporosis in femoral neck [OR: 0.98(0.96,0.99)]. Besides, results suggested that using TNFi in the last 1 year was a protective factor for osteopenia in either the lumbar spine [OR: 0.27 (0.08, 0.84)] or total hip [OR: 0.37 (0.14, 0.93)]. Increased serum uric acid was a risk factor for osteoporosis in total hip [OR: 1.01(1.00, 1.01)].

**Table 3:** Odd ratios of variables from selective models of 3 measurement sites

Detective sites	Osteopenia			Osteoporosis		
	Variables	Odd ratios (95%CI)	*p*	Variables	Odd ratios (95%CI)	*P*
Lumber spine^a^	Age	1.15 (1.10, 1.21)	< 0.001	Age	1.25 (1.18, 1.33)	< 0.001
	BMI	0.88 (0.77, 0.99)	0.038	BMI	0.68 (0.57, 0.79)	< 0.001
	Serum 25(OH)D3 level	0.99 (0.98, 1.00)	0.030	Serum 25(OH)D3 level	0.97 (0.96, 0.98)	< 0.001
	TNFi usage in the last one year	0.27 (0.08, 0.84)	0.027			
Femoral neck^b^	Age	1.17 (1.12, 1.22)	< 0.001	Age	1.26 (1.18, 1.36)	< 0.001
	BMI	0.85 (0.77, 0.95)	0.003	BMI	0.62 (0.50, 0.75)	< 0.001
	Rheumatoid factor concentration	1.00 (0.99, 1.02)	0.183	Serum 25(OH)D3 level	0.98 (0.96, 0.99)	0.002
				sUA	1.00 (1.00, 1.01)	0.068
				Disease duration	1.00 (0.89, 1.13)	0.967
				Serum phosphate level	1.87 (0.48, 29.95)	0.631
Total hip^c^	Age	1.11 (1.08, 1.15)	< 0.001	Age	1.15 (1.10, 1.21	< 0.001
	BMI	0.84 (0.77, 0.92)	< 0.001	BMI	0.68(0.58,0.78)	< 0.001
	TNFi usage in the last one year	0.37 (0.14, 0.93)	0.040	sUA	1.01(1.00, 1.01)	0.001

*BMI* Body mass index, *25(OH)D3* Calcifediol, *TNFi* Tumor necrosis factor-α inhibitor, *sUA* Serum uric acid level

^a^Preselected logistic regression (models A) were optimal for both osteopenia and osteoporosis in lumber spine

^b^LASSO (models B) were optimal for both osteopenia and osteoporosis in femoral neck (R)

^c^Model A and B were optimal for osteopenia and osteoporosis respectively in total hip

## Discussion

Our study detected that in male participants aged at and over 50 and postmenopausal women, the frequency of osteopenia and osteoporosis in the lumbar spine of in-patients with RA was significantly 1.3-fold and 1.2-fold higher than these in healthy counterparts, respectively. The overall frequency of OP in our RA cohort is higher than previous studies, which reported 22.4–46.8% [29, 30]; osteopenia is in the range of the previous reported 25% ~ 34% [7, 8]. Although the significant prevalence of bone loss was only found in the lumbar spine, it was higher than the previous reported (from 31.5 to 36.2%) [3, 4].

The age at which BMD of these sites drops sharply arrived earlier in RA patients, especially in the femoral neck. This point is a noticeable finding of the present study. It suggested that RA patients’ turning points to develop into OPF maybe earlier in the femoral neck. However, in China, the femoral neck is not a routine monitoring choice but the lumbar spine. Overall, these findings suggest that the BMD of the femoral neck also needs appropriate and earlier management than the lumbar spine.

Except for well-documented risk factors (increasing age and lower BMI) consistently associated with the risk of osteopenia and osteoporosis among in-patients with RA, TNFi usage in the last 1 year was found a protective factor for osteopenia in the lumbar spine and total hip by covariates-preselected manually logistic regression. It suggested that using TNFi could not merely reduce disease activity but also protect BMD in the lumber spine and total hip. TNF-α, a key proinflammatory cytokine in RA, can stimulate osteoclastogenesis via RANKL activation [31], leading to systemic bone loss. Since many observational studies had reported TNF-α blockers could either improve or stabilize BMD in the aforementioned measurement sites [14, 32], we forced ‘the usage of TNFi in the last one year before DXA examination’ into the logistic regression models. However, in our study, TNFi usage did not appear to influence BMD of the femoral neck (i.e., was not selected by LASSO models). Actually, even we forced the ‘TNFi usage’ variable into the logistic regression models for osteopenia or OP in the femoral neck, it was not a significant influential factor (data were not showed) for BMD in this site.

As Chen et al. has pointed out, RA patients who received concomitant anti-osteoporotic therapy and bDMARDs would experience a satisfactory BMD preserving effect [33]. However, in the present study, the low using proportion of anti-osteoporotic drugs, including bisphosphonates and Teriparatideacetate in either HC or patients, might be responsible for the inconsistent results. Therefore, though we had forced the ‘overall anti-osteoporotic medication history’ into the logistic regression models, this variable could not significantly influence bone loss in RA and, eventually, did not enter the final sets of logistic regression models.

Sabbagh et al. [34] found that the inadequate Vitamin D status has a considerably strong association with disease activity in RA cases, and active RA with anti-CCP positivity was associated with lower BMD [35]. Our finding was similar partially, which indicated the need for proper evaluation of Vitamin D status in these patients to ensure the intake of the recommended amount of Vitamin D, but positive anti-CCP was not associated with bone loss in our study.

Serum uric acid (sUA) was found as a risk factor for reduced BMD in the total hip of RA patients (11.6% patient with hyperuricemia and an sUA mean value of 314.1 ± 103.9 μmol/L, data were not shown). Several studies have demonstrated that sUA has bilateral effects on bone health. UA is linked to bone loss in hyperuricemia and gout, especially the increased risk of hip fracture [36, 37]. However, UA is the primary antioxidant in human plasma and accounts for more than 60% of the capacity to scavenge free oxidative radicals [38]; thus, it acts as an antioxidant to prevent bone loss and osteoporosis when in the normal physiologic range [39, 40]. Our finding suggested that in RA patients, proper management of hyperuricemia might benefit their hip bone mineral density, lowering the risk of subsequent osteoporotic fracture, which increases the mortality rate [41] and societal and economic cost [42].

This study has limitations. First, we did not specify the current or cumulative steroid dose and disease activity of patients. Second, we cannot exclude the possibility of patient selection bias because the three centers participating in this study were tertiary referral centers in South China. Therefore, BMD measurement rates in this study cannot represent the real rate of DXA in our country. Our analysis also ignored the possible variability that might be caused by different patterns or management of patients across centers. A mixed-effects logistic regression could improve the precision of the estimates. Third, clinicians were more prone to advise in-patients with higher disease activity and longer disease duration and healthy subjects with higher well-documented risks to have DXA examination. Hence, our study revealed a higher prevalence of OP than previously reported. Due to the limitation of sample size and cross-sectional study, a prospective and large-scale follow-up is looked forward to in the future.

In conclusion, RA patients enrolled in the study were more prone and earlier to have bone loss than HC. Our study suggests more attention should be paid to measuring BMD in RA patients aging with lower BMI or hyperuricemia. Besides, serum vitamin D and all three measurement sites are recommended to check routinely. TNFi usage in the last year might benefit bone mass.

## Supplementary Information

**Additional file 1: Figure S1.** Distribution of gender (a) and BMI(b) of patients with RA and HC. *: p for trend with Cochran-Armitage test. **Figure S2.** *: p for trend with Cochran-Armitage test. **Figure S3.** The changing trend of BMD with aging (a) and weight-gaining (b) of patients with RA and HC. **Table S1.** BMD in three detective sites of all participants according to age groups. **Table S2.** DXA results of ‘score below the expected range for age’. **Table S3.** The three approaches development for the model of osteopenia, osteoporosis and their performance.

## Data Availability

Because of the confidentiality of the data used for this study and the strict privacy policy of the Third Affiliated Hospital of Sun Yat-sen University, Zhuhai Hospital of Guangdong Chinese Medicine, Ganzhou Municipal Hospital, and Fujian Provincial Hospital, stating the data be kept among the designated research personnel only. Access to the computer code used in this research is available by request to the corresponding author.

## Abbreviations

RA: Rheumatoid arthritis
BMD: Bone mineral density
OP: Osteoporosis
TNF-α: Tumor necrosis factor-α
RANKL: Receptor activator of nuclear factor kappa B ligand
OPF: Osteoporotic fracture
GCs: Glucocorticoids
BMI: Body mass index
TNFi: TNF inhibitor
HC: Healthy controls
ACR: American college of rheumatology
EULAR: European league against rheumatism
DXA: Dual-energy X-ray absorptiometry
NSAIDs: Non-steroidal anti-inflammatory drugs
anti-CCP: Anti-cyclic citrullinated peptide antibodies
AKA: Antikeratin antibodies
RA33: Anti-RA33 antibodies
SD: Standard error
LASSO: Least absolute shrinkage and selection operator
RF: Random forest
OOB: Out-of-bag
25(OH)D3: Calcifediol
sCr: Serum creatine level
sUA: Serum uric acid
