# Supplementary material for: Prevalence and risk factors for bone loss in rheumatoid arthritis patients from South China: modeled by three methods
Source: BMC Musculoskelet Disord. 2021 Jun 12;22:534. doi: 10.1186/s12891-021-04403-5 (PMC8199806; doi:10.1186/s12891-021-04403-5)
Supplement: Supplementary file 1 — Additional file 1: Figure S1. Distribution of gender (a) and BMI(b) of patients with RA and HC. *: p for trend with Cochran-Armitage test. Figure S2. *: p for trend with Cochran-Armitage test. Figure S3. The changing trend of BMD with aging (a) and weight-gaining (b) of patients with RA and HC. Table S1. BMD in three detective sites of all participants according to age groups. Table S2. DXA results of ‘score below the expected range for age’. Table S3. The three approaches development for the model of osteopenia, osteoporosis and their performance. [file 12891_2021_4403_MOESM1_ESM.docx]

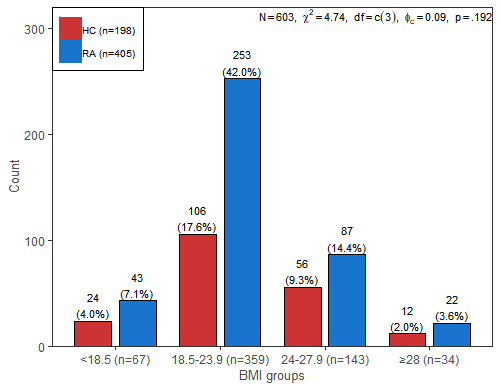

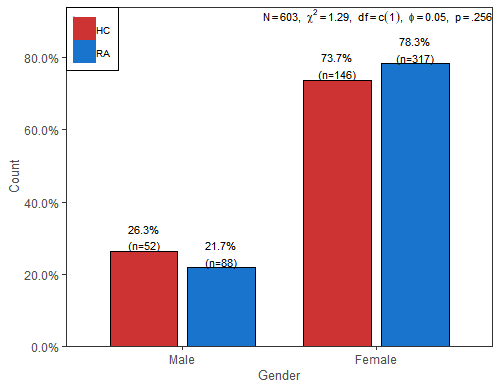

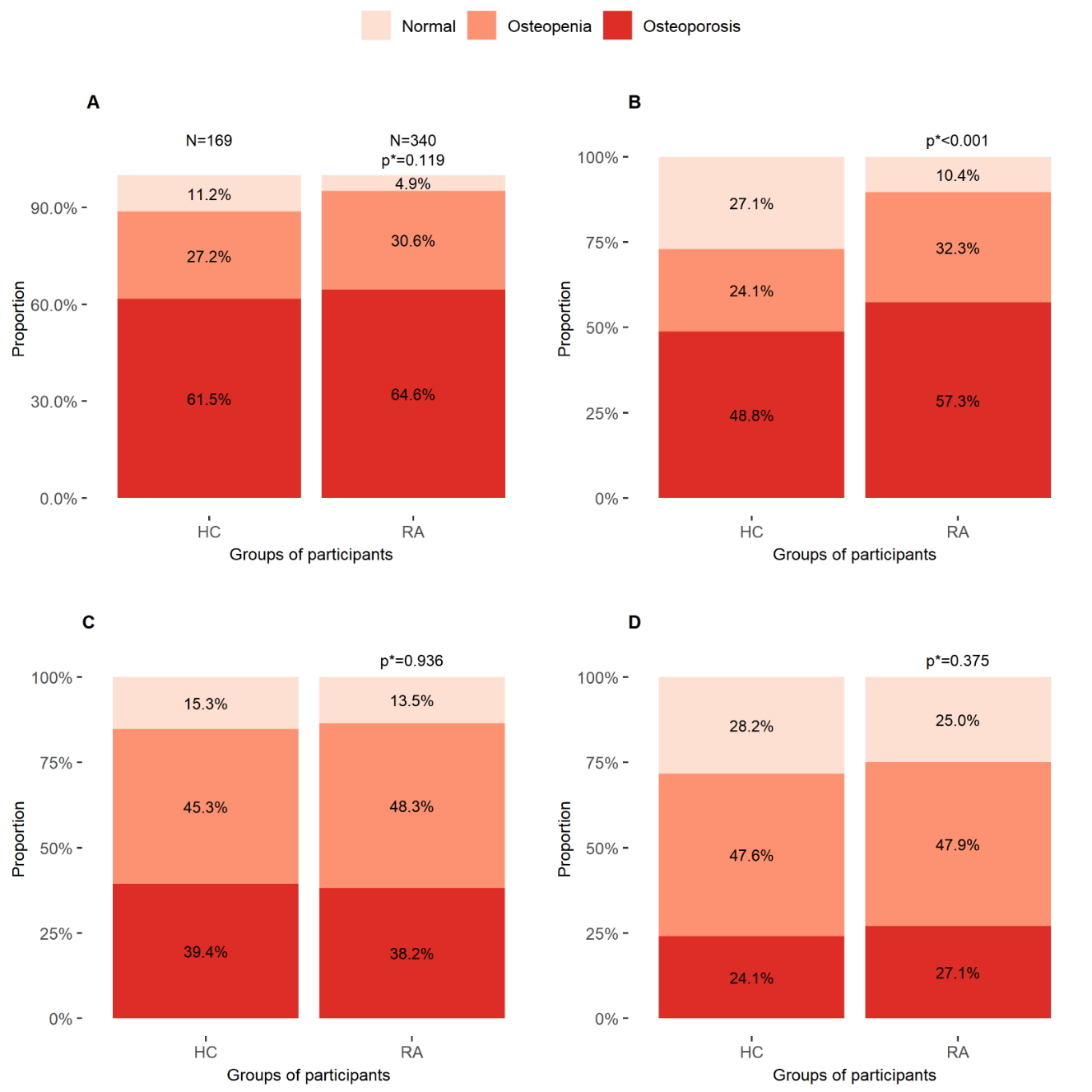


**Supplementary Figure 2.**

***: *p* for trend with *Cochran-Armitage* test.**

**a, b, c, d: the prevalence of bone loss in any detective sites, lumber spine, femoral neck and total hip respectively, in patients with RA and HC**

**Supplementary Figure 1. Distribution of gender (a) and BMI(b) of patients with RA and HC.**

**b**

**a**


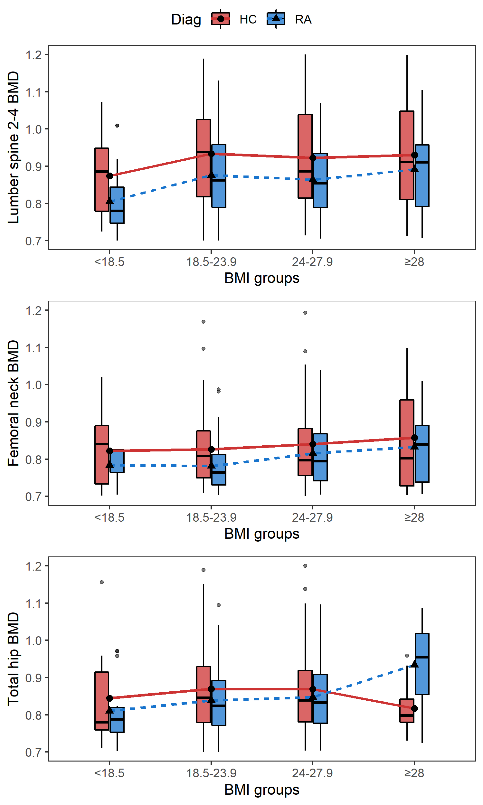

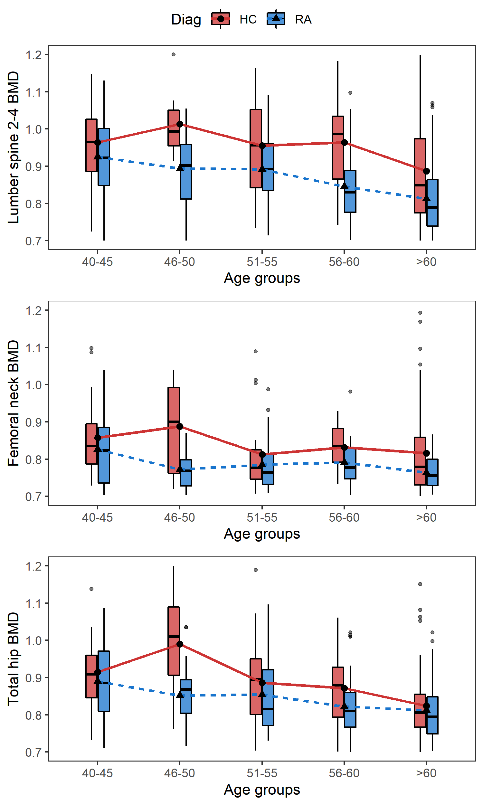


**(a)**

**(b)**

**Supplementary Figure 3 The changing trend of BMD with aging (a) and weight-gaining (b) of patients with RA and HC.**

| **Supplementary Table 1. BMD in three detective sites of all participants according to age groups** | | | | | | | |
| --- | --- | --- | --- | --- | --- | --- | --- |
| **Age groups, years** | **Measurement, n** | **Lumber 2-4 spine** | | **Femoral neck** | | **Total hip** | |
|  |  | **g/cm^2^, mean (SD)** | ***P*** | **g/cm^2^, Mean (SD)** | ***P*** | **g/cm^2^, mean (SD)** | ***P*** |
| 40-45 |  |  | 0.403 |  | 0.014 |  | 0.268 |
| HC | 26 | 0.947 (0.144) |  | 0.827 (0.129) |  | 0.906 (0.167) |  |
| RA | 79 | 0.921 (0.111) |  | 0.751 (0.132) |  | 0.866 (0.121) |  |
| 46-50 |  |  | 0.002 |  | 0.001 |  | 0.047 |
| HC | 11 | 1.042 (0.124) |  | 0.888 (0.122) |  | 0.960 (0.173) |  |
| RA | 50 | 0.883 (0.106) |  | 0.721 (0.078) |  | 0.840 (0.088) |  |
| 51-55 |  |  | <0.001 |  | <0.001 |  | 0.005 |
| HC | 25 | 1.020 (0.183) |  | 0.787 (0.113) |  | 0.891 (0.139) |  |
| RA | 53 | 0.833 (0.149) |  | 0.667 (0.130) |  | 0.790 (0.145) |  |
| 56-60 |  |  | 0.003 |  | 0.008 |  | 0.153 |
| HC | 24 | 0.910 (0.171) |  | 0.726 (0.144) |  | 0.813 (0.150) |  |
| RA | 69 | 0.785 (0.126) |  | 0.634 (0.117) |  | 0.762 (0.135) |  |
| >60 |  |  | <0.001 |  | <0.001 |  | <0.001 |
| HC | 112 | 0.875 (0.189) |  | 0.703 (0.134) |  | 0.768 (0.144) |  |
| RA | 154 | 0.737 (0.127) |  | 0.580(0.110) |  | 0.703 (0.130) |  |
| HC: healthy controls; RA: rheumatoid arthritis | | | | | | | |

| **Supplementary Table 2. DXA results of ‘score below the expected range for age’** | | | |
| --- | --- | --- | --- |
|  | **HC, n=28** | **RA, n=65** | ***P***** |
| **Detective sites, n (%)** |  |  |  |
| Any site | 3 (10.7) | 13 (20.0) | 0.375 |
| Lumber spine | 2 (7.1) | 10 (15.4) | 0.336 |
| Femoral neck | 3 (10.7) | 9 (13.9) | 0.859 |
| Total hip | 3(10.7) | 6 (9.2) | 0.963 |
| *P***: *Fisher’s* exact test | | | |

| **Supplementary Table 3.** **The three approaches development for the model of osteopenia, osteoporosis and their performance** | | | | | |
| --- | --- | --- | --- | --- | --- |
| **Approaches** | **Variables (n)** | **Variables** | **C-statistics (95%CI)** | **Calibration slope (95%CI)** | **Accuracy*** |
| **Osteopenia** |  |  |  |  |  |
| **Lumber spine 2-4** | | | | |  |
| *Model A*: clinical knowledge-based | 4 | age; BMI; serum 25(OH)D3 level; TNFi usage in the last one year | 0.81(0.67-0.94) | 1.01(0.54-1.49) | 82.3 |
| *Model B*: statistics-driven (LASSO) | 1 | age | 0.84(0.73-0.95) | 0.88(0.35-1.40) | 74.5 |
| *Model C*: random forest | 9 | age; BMI; serum 25(OH)D3 level; sUA; sCr; serum calcium level; rheumatoid factor concentration; CH50 level; CRP level | 0.79(0.66-0.92) | 0.96(0.72-1.20) | 78.4 |
| **Femoral neck** | | | | | |
| Model A: clinical knowledge-based | 3 | age; BMI; rheumatoid factor concentration | 0.79(0.68-0.90) | 0.97(0.62-1.31) | 72.1 |
| Model B: statistics-driven (LASSO) | 3 | age; BMI; rheumatoid factor concentration | 0.79(0.68-0.90) | 0.97(0.62-1.31) | 75 |
| Model C: random forest | 9 | age; BMI; rheumatoid factor concentration; sUA; sCr; CH50 level; serum phosphate level; anti-CCP concentration; CRP level | 0.75(0.63-0.88) | 0.96(0.73-1.20) | 76.5 |
| **Total hip** | | | | | |
| Model A: clinical knowledge-based | 3 | age; BMI; TNFi usage in the last one year | 0.76(0.64-0.87) | 1.07(0.62-1.51) | 71.8 |
| Model B: statistics-driven (LASSO) | 5 | age; BMI; disease duration; types of cDMARDs recently taking; ANA titer | 0.73( 0.62-0.85) | 1.06(0.62-1.51) | 66.7 |
| Model C: random forest | 10 | age; BMI; sCr; sUA; disease duration; serum 25(OH)D3 level; CRP level; ESR; anti-CCP concentration; rheumatoid factor concentration | 0.70(0.57-0.82) | 1.01(0.63-1.40) | 71.8 |
| **Osteoporosis** | | | | | |
| **Lumber spine 2-4** | | | | | |
| Model A: clinical knowledge-based | 3 | age; BMI; serum 25(OH)D3 level | 0.95(0.89-0.99) | 0.99(0.77-1.22) | 91.8 |
| Model B: statistics-driven (LASSO) | 5 | age; BMI; serum 25(OH)D3 level; anti-CCP concentration†; chronic GC usage | 0.93(0.87-0.98) | 1.00(0.91-1.09) | 89 |
| Model C: random forest | 7 | age; BMI; serum 25(OH)D3 level; sUA; sCr; ESR; CRP level | 0.92(0.86-0.99) | 1.04(0.89-1.18) | 89 |
| **Femoral neck** | | | | | |
| Model A: clinical knowledge-based | 3 | age; BMI; rheumatoid factor concentration | 0.92(0.85-0.99) | 1.03(0.77-1.30) | 85 |
| Model B: statistics-driven (LASSO) | 2 | age; BMI | 0.93(0.85-0.99) | 1.03(0.78-1.27) | 90 |
| Model C: random forest | 6 | age; BMI; disease duration; sUA; serum 25(OH)D3 level; serum phosphate level | 0.95(0.90-0.99) | 1.02(0.98- 1.07) | 86.7 |
| **Total hip** | | | | | |
| Model A: clinical knowledge-based | 3 | age; BMI; disease duration | 0.93(0.86-0.99) | 1.01(0.66-1.36) | 76.7 |
| Model B: statistics-driven (LASSO) | 3 | age; BMI; sUA | 0.93(0.87-0.99) | 1.02(0.68-1.37) | 76.8 |
| Model C: random forest | 5 | age; BMI; disease duration; sUA; ESR; | 0.87(0.78-0.96) | 1.02(0.67- 1.38) | 73.3 |
| †: Removed from the multivariate regression model due to p-value greater than 0.05.  *: accuracy of internal verification via calculating the confusion matrix | | | | | |

c
